# A practical guide for fast implementation of SNARE-mediated liposome fusion

**DOI:** 10.52601/bpr.2023.230017

**Published:** 2024-02-29

**Authors:** Shen Wang, Cong Ma

**Affiliations:** 1 Key Laboratory of Molecular Biophysics of the Ministry of Education, College of Life Science and Technology, Huazhong University of Science and Technology, Wuhan 430074, China

**Keywords:** Liposome fusion, SNARE, *In-vitro* reconstitution, Protocol

## Abstract

Soluble N-ethylmaleimide-sensitive factor attachment protein receptor (SNAER) family proteins are the engines of most intra-cellular and exocytotic membrane fusion pathways (Jahn and Scheller 2006). Over the past two decades, *in-vitro* liposome fusion has been proven to be a powerful tool to reconstruct physiological SNARE-mediated membrane fusion processes (Liu *et al.* 2017). The reconstitution of the membrane fusion process not only provides direct evidence of the capability of the cognate SNARE complex in driving membrane fusion but also allows researchers to study the functional mechanisms of regulatory proteins in related pathways (Wickner and Rizo 2017). Heretofore, a variety of delicate methods for *in-vitro* SNARE-mediated liposome fusion have been established (Bao *et al.* 2018; Diao *et al.* 2012; Duzgunes 2003; Gong *et al.* 2015; Heo *et al*. 2021; Kiessling *et al.* 2015; Kreye *et al.* 2008; Kyoung *et al.* 2013; Liu *et al.* 2017; Scott *et al.* 2003). Although technological advances have made reconstitution more physiologically relevant, increasingly elaborate experimental procedures, instruments, and data processing algorithms nevertheless hinder the non-experts from setting up basic SNARE-mediated liposome fusion assays. Here, we describe a low-cost, timesaving, and easy-to-handle protocol to set up a foundational *in-vitro* SNARE-mediated liposome fusion assay based on our previous publications (Liu *et al.* 2023; Wang and Ma 2022). The protocol can be readily adapted to assess various types of SNARE-mediated membrane fusion and the actions of fusion regulators by using appropriate alternative additives (*e*.*g*., proteins, macromolecules, chemicals, *etc*.). The total time required for one round of the assay is typically two days and could be extremely compressed into one day.

## INTRODUCTION

Membrane-enclosed organelles and vesicles communicate with one another via membrane contact and membrane fusion for the exchange and transport of proteins, lipids, and metabolites in all eukaryotic cells (Bonifacino and Glick 2004; Jahn *et al.* 2003; Wickner 2010). SNARE family proteins are the engines of most types of membrane fusion events (Jahn and Scheller 2006). For instance, neurotransmitter release, where synaptic vesicle fuses with the presynaptic membrane, is mediated by the Q_a_-SNARE Syntaxin-1 (Syx1) and Q_bc_-SNARE SNAP-25 (SN25) residing on presynaptic membrane and the R-SNARE VAMP2 (also known as Synaptobrevin-2, Syb2) resided on synaptic vesicle membrane (Jahn and Fasshauer 2012). The three SNAREs assemble into the SNARE complex and bring the opposite membrane into close proximity to overcome membrane repulsion and drive membrane fusion (Chen and Scheller 2001; Sudhof and Rothman 2009). *Choanoflagellates*, one of the simplest eukaryotic taxa, possess over 20 SNARE genes, while the number is up to 30 among yeasts and 40 among mammals (Kloepper *et al.* 2007, 2008). Therefore, SNAREs are believed to undergo several rounds of gene duplication in evolution, which leads to apparent redundancy in specific membrane fusion processes. The redundancy and variable subcellular localizations of SNAREs thus hinder one from analyzing the molecular function of SNAREs through conventional cytogenetics and cell biology approaches. *In-vitro* liposome fusion is a powerful tool to reconstruct physiological SNARE-mediated membrane fusion processes (Liu *et al.* 2017). The reconstitution of the membrane fusion process not only provides direct evidence of the capability of the cognate SNARE complex in driving membrane fusion but also allows researchers to study the functional mechanisms of other regulatory proteins in related pathways.

Over the past two decades, *in-vitro* reconstitution of SNARE-mediated membrane fusion has been well developed (Bao *et al.* 2018; Diao *et al.* 2012; Duzgunes 2003; Gong *et al.* 2015; Heo *et al.* 2021; Kiessling *et al.* 2015; Kreye *et al.* 2008; Kyoung *et al.* 2013; Liu *et al.* 2017; Scott *et al.* 2003). However, increasingly elaborate experimental procedures, instruments, and data processing algorithms also hinder the non-experts from setting up foundational SNARE-mediated liposome fusion assays. Here, we provide a low-cost, time-saving, and easy-to-handle protocol that is based on our previous publications (Liu *et al.* 2023; Wang and Ma 2022). We believe that the protocol would enable non-experts to quickly set up their *in-vitro* SNARE-mediated liposome fusion assays and further analyze the actions of proteins of interest in the process.

## DEVELOPMENT OF SNARE-MEDIATED LIPOSOME FUSION

**Figure 1:**
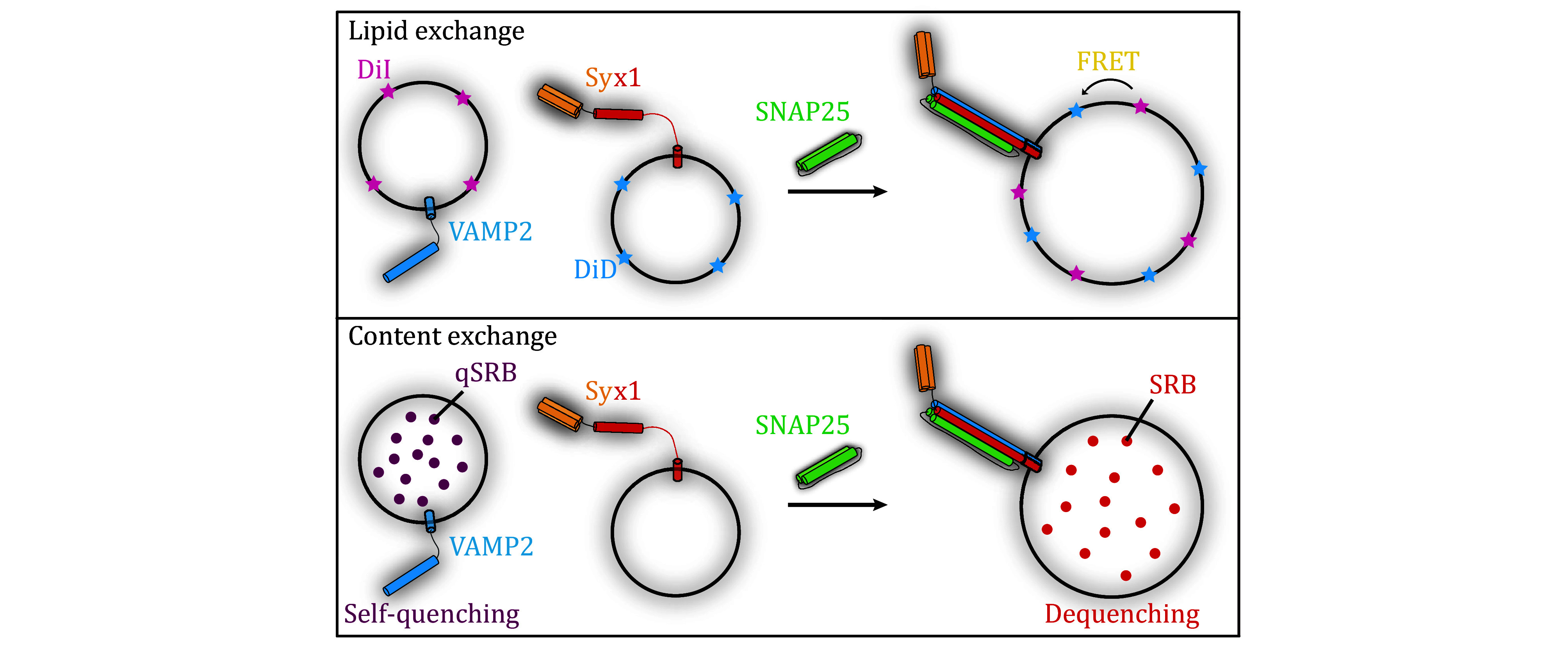
Illustration of SNARE-mediated liposome fusion assays. Upper panel: lipid exchange tracked by FRET between DiI and DiD in SNARE-mediated liposome fusion assay. Lower panel: content exchange tracked by dequenching of sulforhodamine B (SRB) in SNARE-mediated liposome fusion assay

Techniques for *in-vitro* reconstitution of SNARE-mediated membrane fusion could be traced back to the 1990s after the discovery of neuronal SNAREs Syx1, SN25, and VAMP2 (Nickel *et al.* 1999; Weber *et al.* 1998). Membrane fusion can be tracked by two successive steps, *i*.*e*., lipid exchange and content exchange (Fig. 1). The former usually utilizes florescent lipids that are incorporated into liposomes. The fluorescent lipids constitute a FRET pair, thus lipid exchange between opposite membranes during liposome fusion could be tracked by an arbitrary fluorimeter equipped with specific filters or monochromators. Frequently used fluorescent lipids as FRET pairs are listed in Table 1. While content exchange is more physiologically significant since lipid exchange sometimes occurs in hemi-fusion. Content exchange could be tracked by fluorescence signals as well, but the mechanisms vary from specific reagents applied in the system (Table 1). Meanwhile, simultaneous detection of lipid and content exchange in liposome fusion has been introduced in recent literature (Camacho *et al.* 2021; Quade *et al.* 2019; Song *et al.* 2021; Song and Wickner 2021; Stepien *et al.* 2019). Furthermore, the development of single-molecule techniques makes it possible to track single-liposome fusion events within a sub-second time scale (Bao *et al.* 2018; Diao *et al.* 2012; Heo *et al.* 2021; Kyoung *et al.* 2013; Kiessling *et al.* 2015; Lai *et al.* 2017).

**Table 1:** Frequently-used indicators in *in-vitro* liposome fusion assays

Type	Reagents	Mechanism	Reference
Lipid exchange	NBD-PE/Rhodamine-PE	FRET	Weber *et al*. 1998
	DiI/DiD	FRET	Diao *et al.* 2017
	Marina Blue-PE/NBD-PE	FRET	Liu *et al*. 2017; Song and Wickner 2021
Content exchange	^33^P-DNA/biotin-DNA pair	Autoradiography	Nickel *et al.* 1999
	Sulforhodamine B (SRB)	Self-qunching/dequnching	Wang *et al.* 2016
	Tb^3 + ^/DPA	Terbium luminescence	Duzgunes 2003
	CB[7]-Cy3/Ad-Cy5	FRET	Gong *et al.* 2015
	PhycoE-biotin/Cy5-streptavidin	FRET	Liu *et al.* 2017; Song and Wickner 2021

Although technological advances have made *in-vitro* liposome fusion more physiologically relevant, increasingly elaborate experimental procedures, instruments, and data processing algorithms nevertheless hinder the non-experts from setting up basic SNARE-mediated liposome fusion assays. This protocol is designed to present a detailed procedure for fast implementation of foundational *in-vitro* SNARE-mediated liposome fusion. The method is proven to be efficient and stable, which can be readily adapted to assess various types of SNARE-mediated membrane fusion and the actions of fusion regulators by using appropriate alternative additives (*e*.*g*., proteins, macromolecules, chemicals, *etc*.). The total time required for one round of the assay is typically two days and could be extremely compressed into one day.

## OVERVIEW OF THE PROTOCOL

Lipids dissolved in an aqueous solution that contains the proper type and concentration of detergents could spontaneously form liposomes if the concentration of detergents was diluted lower than their critical micelle concentration (CMC) (Ollivon *et al.* 2000). The sizes of liposomes could be variable based on the lipids with different fatty acid chains (length and saturation) and headgroup sizes. SNAREs with the transmembrane domain are purified with the same detergents and supplied to the lipid-detergent mixtures. The protocol adopts a fast-desalting method to prepare proteoliposomes (Wang and Ma 2022). Briefly, the lipid-detergent-protein mixtures are incubated at room temperature for 20 min. The mixtures are then loaded into the Sephadex G-25 desalting column. Colored fractions (liposomes containing fluorescent lipids) were collected and pooled. Qualities of the prepared proteoliposomes are measured by dynamic light scattering (DLS). In liposome fusion assay, t-liposome harboring the Q_a_-SNARE (Syx1) and v-liposome harboring the R-SNARE (VAMP2) are mixed with equivalent concentration (~100 μmol/L), SN25 is supplied to 5 μmol/L. The reaction is carried out with a fluorescence plate reader equipped with proper excitation and emission wavelengths. Note that the concentration of SNAREs used in the assay will likely need to be adapted and optimized.

## APPLICATIONS AND ADVANTAGES OF THE PROTOCOL

This protocol is simplified from previous lengthy methods, retaining the most fundamental steps and enabling one to quickly set up SNARE-mediated liposome fusion assay with the lowest costs. The results are stable and have good repeatability. Importantly, the streamlined steps effectively reduce the overall time, allowing for large-scale screening in a short timeframe. It can be applied to high-throughput experiments when paired with appropriate instruments.

## LIMITATIONS OF THE PROTOCOL

This protocol has two major limitations. Since only lipid exchange is tracked during liposome fusion, one could not confirm whether the signal is given by hemi-fusion or full fusion. In addition, the signals of liposome fusion are based on average signals given by large ensemble, which could not present effective information (kinetics and thermodynamics) for individual fusion events.

## MATERIALS AND EQUIPMENT

### Reagents

• 2-amino-2-(hydroxymethyl)-1,3-propanediol (Tris base, BioFoxx, #1115GR550)

• NaCl (Sinopharm Chemical Regent Co. Ltd., #10019308)

• Ethanol (Sinopharm Chemical Regent Co. Ltd., #10009218)

**[CAUTION!]** Ethanol is toxic and flammable. Avoid flames and handle in a chemical hood.

• Chloroform (Sinopharm Chemical Regent Co. Ltd., #10006818)

**[CAUTION!]** Chloroform is toxic and carcinogenic. All sample preparations involving chloroform should be performed under a chemical hood.

• HCl (Sinopharm Chemical Regent Co. Ltd., #10011018)

**[CAUTION!]** HCl is highly corrosive and volatile. Chemical hood and personal protective equipment are required while handling.

• n-Octyl-β-D-glucopyranoside (beta-OG, Sangon Biotech, #A607057)

• Tris(2-carboxyethyl)phosphine hydrochloride (TCEP, Sigma, #C4706)

• 1-palmitoyl-2-oleoyl-glycero-3-phosphocholine (POPC, Avanti Polar Lipids, # 850457P)

• 1,2-dioleoyl-sn-glycero-3-phospho-L-serine sodium salt (DOPS, Avanti Polar Lipids, #840035P)

• 1,1'-dioctadecyl-3,3,3',3'-tetramethylindodicarbocyanine perchlorate (DiD; DiIC18(5), Invitrogen, #D307)

• 1,1'-dioctadecyl-3,3,3',3'-tetramethylindocarbocyanine perchlorate (DiI; DiIC18(3), Invitrogen, #D282)

**[CAUTION!]** Powdered lipids are harmful to skin, respiratory tract, and eyes. Personal protective equipment is required while handling.

### Reagent setup

#### TCEP stock solutions

Dissolve 1.43 g TCEP in 10 mL Milli-Q water. The molar concentration of the TCEP stock solution is 0.5 mol/L. Aliquot the solution into 1.5 mL microcentrifuge tubes. The TCEP stock solution can be stored for one year at –20 °C.

#### Lipid stock solutions

Dissolve 100 mg POPC/DOPS into a glass tube with 10 mL chloroform. Dissolve 5 mg DiD/DiI into a glass tube with 5 mL ethanol. Aliquot the dissolved lipids into 9-mm amber glass screw thread vials, screw with solid top PTFE-lined cap and seal with parafilm. The lipid stock solutions can be stored for one year at –20 °C.

**[CAUTION!]** Chloroform is toxic and carcinogenic. All sample preparations involving chloroform should be performed under a chemical hood.

**[CAUTION!]** Ethanol is toxic and flammable. Avoid flames and handle in a chemical hood.

#### Buffer A (50 mmol/L Tris-Cl pH 7.5, 150 mmol/L NaCl, 0.5 mmol/L TCEP, 50 mmol/L beta-OG)

Dissolve 0.30 g Tris base, 0.44 g NaCl, 0.73 g beta-OG in 45 mL Milli-Q water. Add 50 μL 0.5mol/L TCEP. Adjust the pH to 7.5 with HCl. Bring the volume to 50 mL. This solution can be stored for one month at 4 °C and one year at –20 °C.

#### Buffer B (50 mmol/L Tris-Cl pH 7.5, 150 mmol/L NaCl, 0.5 mmol/L TCEP)

Dissolve 3.03 g Tris base and 4.38 g NaCl in 450 mL double-deionized water. Add 0.5 mL 0.5mol/L TCEP. Adjust the pH to 7.5 with HCl. Bring the volume to 500 mL. This solution can be stored for three months at 4 °C.

**[CRITICAL STEP]** Always supply TCEP before adjusting the pH of the solution, since TCEP can drastically lower the pH of the solution.

### Equipment

• Glass tubes Φ15 × 100 (CITOGLAS, #84004-0125)

• Beakers, 1L (CITOGLAS, #84000-1000)

• Graduated cylinders, 1L (CITOGLAS, #84205-1181)

• Reagent bottles, 500 mL (Haimen Shengbang Laboratory Equipment Co. Ltd., #HX-G04)

• 9-mm amber glass screw thread vials (Thermo Scientific, #033919)

• Solid top PTFE-lined cap (Thermo Scientific, #B7800-1-9)

• Parafilm (Fisher Scientific, #13-374-10)

• Milli-Q water purification system (Barnstead)

• Microcentrifuge tubes, 1.5 mL (Axygen, #MCT-150-C)

• Centrifuge tubes, 50 mL, polypropylene (Corning, #430291)

• 96-well ELISA plate (Wuxi NEST Biotechnology Co. Ltd., # 514201)

• Sephadex G-25 desalting column, prepacked 8.3 mL (GE Healthcare, #17-0851-01)

• Nitrogen gas, steel cylinder (Wuhan Shuanglong)

• Pipetman (P2, P20, P200 and P1000; Gilson, #F144801, #F123600, #F123601, #F123602)

• Sorvall Legend Micro 17 Microcentrifuge (Thermo Scientific, #75002403)

• VORTEX-5 vortex mixer (Haimen Kylin-Bell Lab Instruments Co. Ltd.)

• Oerlikon Leybold D80 vacuum pump (Oerlikon Leybold, #31046)

• Vacuum desiccator (Shanghai Blupard Instruments Co. Ltd., #PH-010(A))

• The DynaPro NanoStar (Wyatt Technology)

• FluoDia T70 fluorescence plate reader (PTI) equipped with 40-Watt halogen lamp, 530/10BP excitation filter, 580/10BP and 667/10BP emission filters.

## PROCEDURE

The procedure described below uses neuronal SNAREs Syx1, SN25, and VAMP2 (Syb2), the canonical cognate SNAREs, to carry out liposome fusion assay. Expression and purification of these proteins have been described earlier (Wang and Ma 2022). Lipids constitute the liposomes containing the basic phospholipids POPC, DOPS, and fluorescent lipids DiD (as FRET acceptor), DiI (as FRET donor). Alternatively, one may need additional lipids for specific conditions. The usage of other lipids that are not described here should consult the manufactory’s instructions and will likely need to be adapted and optimized.

### Set up of lipid stock solutions [TIMING ~0.5 h]

1 Take the powdered lipids from the –20 °C refrigerator, and bring them back to room temperature.

**[CRITICAL STEP]** In a relatively humid environment, dried powders may absorb water in the air if the temperature of the reagent is lower than the environment temperature.

2 Dissolve 100 mg POPC/DOPS into a glass tube with 10 mL chloroform.

3 Dissolve 5 mg DiD/DiI into a glass tube with 5 mL ethanol.

4 Aliquot the dissolved lipids into 9-mm amber glass screw thread vials, screw with solid top PTFE-lined cap and seal with parafilm. The lipid stock solutions can be stored for one year at –20 °C.

**[CAUTION!]** Chloroform is toxic and carcinogenic. The experiment should be performed under a chemical hood.

**[CAUTION!]** Ethanol is toxic and flammable. Avoid flames and handle in a chemical hood.

### Lipid drying [TIMING variable (at least 4 h)]

5 Follow option A to mix lipids with the proper molar ratio for t-liposomes; follow option B to mix lipids with the proper molar ratio for v-liposomes. The detailed amounts of the lipid mixtures are listed in Table 2.

**Table 2:** The detailed amounts of lipids used for liposome preparation

Lipids	Stock concentration ((mg/mL)/ (mmol/L))	Volume (μL)	
		T-liposome (2.5 μmol)	V-liposome (2.5 μmol)
POPC	10.0 / 13.16	148.2	148.2
DOPS	10.0 / 12.34	40.5	40.5
DiD	1.0 / 1.04	48.1	-
DiI	1.0 / 1.07	-	46.7

(A) T-liposome, 2.5 μmol total lipids, POPC∶DOPS∶DiD = 78%∶20%∶2%. Mix 148.2 μL POPC stock solution, 40.5 μL DOPS stock solution, and 48.1 μL DiD stock solution into a glass tube.

(B) V-liposome, 2.5 μmol total lipids, POPC∶DOPS∶DiI = 78%∶20%∶2%. Mix 148.2 μL POPC stock solution, 40.5 μL DOPS stock solution, and 46.7 μL DiI stock solution into a glass tube.

**[CAUTION!]** Use aerosol barrier tips or microdispensers with positive replacement glass capillaries for transferring the lipid solutions.

**[? TROUBLESHOOTING]**

6 Blow-dry the lipid mixtures carefully under a gentle nitrogen flow. Tilt and rotate the glass tube continuously until a thin film is formed on the wall near the bottom of the tube.

**[CAUTION!]** Chloroform is toxic and carcinogenic. The experiment should be performed under a chemical hood.

**[CRITICAL STEP]** Some specific lipids have high phase-transition temperatures, which may cause precipitation during drying. If that is the case, use a water bath at a temperature higher than the phase-transition temperature when drying the lipid mixtures.

**[? TROUBLESHOOTING]**

7 Keep the tubes in the light-avoiding vacuum desiccator to thoroughly dry the lipid film for at least three hours.

**[PAUSE POINT]** The time for vacuum drying could be variable, which depends on the efficiency of the vacuum desiccator. In our setup, 3-hour vacuuming is enough. For the sake of reliability, long-term vacuuming (12 h) is also recommended.

### Liposome preparation [TIMING ~1 h]

8 Add 400 μL Buffer A to the dried lipid films. Vortex the mixtures vigorously for 5 min until the solution is clear.

**[CRITICAL STEP]** The concentration of beta-OG in buffer A is 50 mmol/L, which is 2-fold than its CMC (20–25 mmol/L). It is suitable to dissolve lipid films at this concentration, however, a higher concentration may be required for higher lipid concentrations.

**[? TROUBLESHOOTING]**

9 Follow option A to reconstitute Syx1 into t-liposomes; follow option B to reconstitute VAMP2 into v-liposomes. The protein-to-lipid ratios of t- and v-liposome are both 1:500.

(A) Mix 5 nmol purified Syx1 full-length proteins with a lipid-detergent mixture containing POPC, DOPS, and DiD. Transfer the mixtures into a 1.5-mL microcentrifuge tube and incubate in the dark at room temperature for 20 min.

(B) Mix 5 nmol purified VAMP2 full-length proteins with a lipid-detergent mixture containing POPC, DOPS, and DiI. Transfer the mixtures into a 1.5-mL microcentrifuge tube and incubate in the dark at room temperature for 20 min.

**[CRITICAL STEP]** The final volume of the protein-lipid-detergent mixtures should not exceed the maximum capacity of the desalting column. The optimal volume is less than 1 mL and typically 0.5 mL for an 8.3-mL Sephadex G-25 column. Note that the concentrations of beta-OG in Buffer A and purified SNAREs are the same (50 mmol/L). The concentration of beta-OG in the final volume should be at least higher than 1.5-fold of its CMC ( ~34 mmol/L).

**[? TROUBLESHOOTING]**

10 Equilibrate the Sephadex G-25 desalting column for 5 CV with Buffer B.

11 Load the protein-lipid-detergent mixtures into Sephadex G-25 desalting column. Colored fractions (liposomes harboring fluorescent lipids) are collected and pooled into 1.5 mL microcentrifuge tubes.

**[CRITICAL STEP]** Typically, for an 8.3-mL Sephadex G-25 desalting column, a 0.5-mL sample will yield ~2.5 mL final fraction. Therefore, the concentration of prepared liposomes is ~1 mmol/L (total lipids). We recommend using the liposomes as soon as possible, since long-term storage may lead to degradation of proteins and oxidation of lipids.

**[PAUSE POINT]** The prepared liposomes can be stored at 4 °C in the dark for 24 h. Alternatively, they can be flash-frozen as small aliquots in liquid nitrogen. Flash-frozen liposomes can be stored in liquid nitrogen or at –80 °C for three months but with activity loss during freezing-thawing cycles.

### Quality control [TIMING ~0.5 h]

12 Take some prepared t- and v-liposomes and dilute them to a concentration of ~20–40 μmol/L (total lipids) with Buffer B.

**[CRITICAL STEP]** Typical DLS instrument utilizes a near-infrared laser as a light source, which is overlapped with the excitation spectrum of DiD. The scattering signal of liposomes harboring DiD with higher concentration may disturbed by DiD fluorescence.

**[? TROUBLESHOOTING]**

13 Perform dynamic light scattering (DLS) analysis. The typical characteristics of the proteoliposomes should range from 40 to 100 nm in diameter and be monodisperse. Alternatively, it is advisable to analyze the liposomes by electron microscopy.

**[? TROUBLESHOOTING]**

### FRET assay to track liposome fusion [TIMING 1–2 h]

14 100 μmol/L t-liposome harboring Syx1 and 100 μmol/L v-liposome harboring VAMP2 are mixed with the addition of 5 μmol/L SN25 to a final volume of 70 μL with Buffer B in the well of a standard 96-well plate.

**[? TROUBLESHOOTING]**

15 Set a parallel negative control group that omits the addition of SN25: mixing 100 μmol/L t-liposome harboring Syx1 and 100 μmol/L v-liposome harboring VAMP2 to a final volume of 70 μL with Buffer B in the well of a standard 96-well plate.

**[CRITICAL STEP]** Negative control is indispensable. Alternatively, one can add the excess cytoplasmic domain of VAMP2 (cdVAMP2) into the reaction described in Step 14 as the negative control.

**[? TROUBLESHOOTING]**

16 Launch the FluorDia T70 fluorescence plate reader, and let it warm up for 10 min.

17 Set 530/10BP as excitation filter, 580/10BP (for DiI) and 667/10BP (for DiD) as emission filters. The scanning dwell time for each well is set to 300 ms. The time spacing for each scanning cycle is set to 30 s (*i*.*e*., data is collected every 30 s).

**[CRITICAL STEP]** Note that time spacing for one scanning cycle depends on the dwell time for each well and the sum of operational wells. In the above setup, 30 s is suitable for up to 24 wells with 300-ms dwell time.

**[? TROUBLESHOOTING]**

### Data processing and visualization [TIMING 0.5–1 h]

18 Divide the summary of fluorescent intensities of DiD and DiI into the fluorescent intensity of DiD for each time point. This will calculate the raw FRET efficiency, proximity ratio (*E*_PR_), between DiD and DiI, which denotes liposome fusion signal:

$ \qquad{E}_{PR}=\frac{{I}_{DiD}}{{I}_{DiD} + {I}_{DiI}} . $

19 Normalize the proximity ratio (*E*_PR_) to the value of the first time point (*t* = 0):

$\qquad {nE}_{PR}\left(t\right)={E}_{PR}\left(t\right)-{E}_{PR}\left(0\right) . $

20 Plot time-*nE*_PR_ traces for each group using Microsoft Excel, GraphPad Prism, Origin, or other equivalent software. One can utilize multiple ways to analyze the data quantitatively. (1) The *nE*_PR_ values at the end of the reaction inform the overall extent of liposome fusion, which is the common visualization approach to assess the thermodynamics of fusion events for different groups; (2) one can also fit the time-*nE*_PR_ traces with single or multiple exponential association function:

$ \qquad f\left(t\right)={f}_{0} + {\sum }_{n\;=\;1}^{n\;\in\; {N}^{ + }}(1-{e}^{-t/{\tau }_{n}}) , $

the time constant (*τ*) or rate constant (*k*, equal to 1/*τ*) will present kinetic information.

**[? TROUBLESHOOTING]**

Troubleshooting advice can be found in Table 3.

**Table 3:** Troubleshooting table

Step	Problem	Possible reason	Solution
5, 6	Cloudy lipid mixtures	(1) Some specific lipids have high phase-transition temperature. (2) Oxidation of unsaturated lipids	(1) Blow-dry the lipids in 45 °C water bath. (2) Purchase new lipids
8, 9	Incomplete dissolution of lipid films	Too much lipids and/or insufficient detergents in dissolution buffer	Supply more Buffer A; check the concentration of detergent in Buffer A
12, 13	No liposomes or liposome size less than 20 nm	(1) Detergents are not removed thoroughly. (2) Inappropriate detergents	(1) Decrease the volume of the samples loaded into desalting column. (2) Use suitable detergents
12, 13	Large particles (>500 nm)	Dirty cuvette	Clean the cuvette thoroughly before analysis
14, 17	Low fusion efficiency	(1) Low liposome concentration. (2) Insufficient functional SNARE proteins reconstituted on the liposomes (degradation or incorrect concentrations) (3) Non-functional SNAREs. (4) Poor lipid quality	(1) Reconfirm the concentrations of liposomes used in the assay. (2) Perform SDS-PAGE to check both the reconstituted liposomes and the SNARE stocks. Purify new proteins and add protease inhibitors during storage. Supply protease inhibitors in liposome fusion assays. (3) Check SNARE complex assembly in detergents. Try refresh the proteins or use truncated form that contains minimal SNARE domain and transmembrane domain. (4) Use freshly prepared proteoliposomes or purchase new lipids
15, 17	Negative control gives robust liposome fusion signal	Poor lipid quality	Use freshly prepared proteoliposomes or purchase new lipids

**[TIMING]**

Steps 1–4, preparation of lipid stock solutions: 0.5 h

Steps 5–7, drying of lipids: 4 h

Steps 8–11, liposome preparation: ~1 h

Steps 12–13, quality analysis of prepared liposomes: ~0.5 h

Steps 14–17, FRET assay to track liposome fusion: 1–2 h

Steps 18–20, data processing and visualization: 0.5–1 h

## ANTICIPATED RESULTS

**Figure 2:**
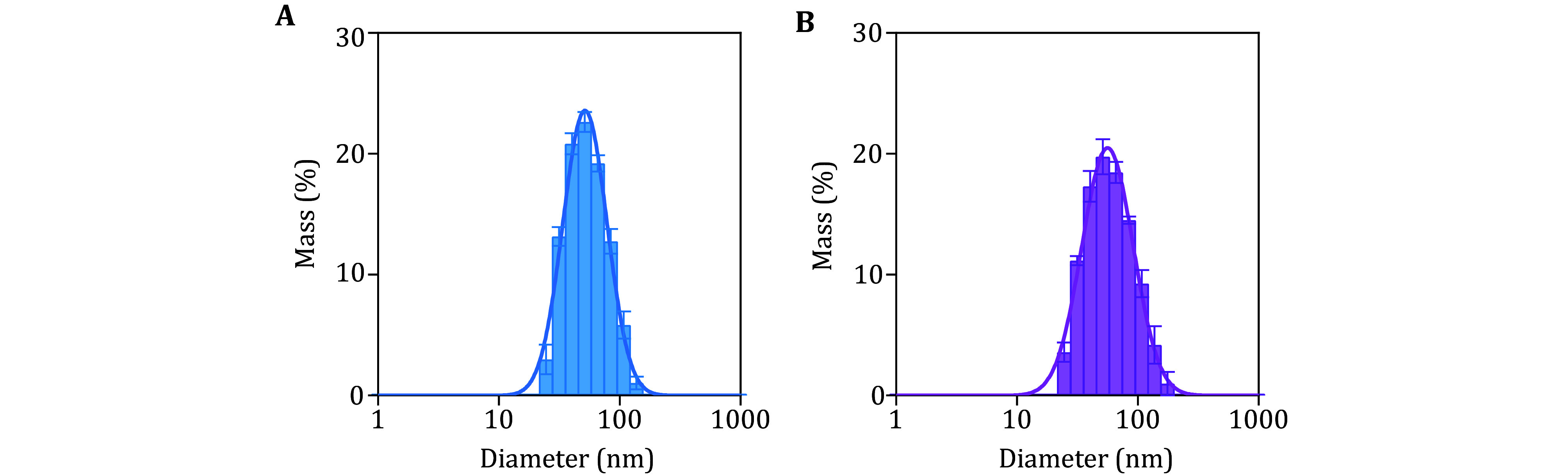
Quality control of the prepared proteoliposomes. Size distributions of t-liposomes reconstituted with Syx1 (**A**) and v-liposomes reconstituted with VAMP2 (**B**) as measured by dynamic light scattering. Histograms are the average of five independent experiments. Error bars indicate standard deviation (SD). The histograms are fitted with the Gaussian function

The typical characteristics of the prepared proteoliposomes should range from 40 to 100 nm in diameter and be monodisperse. With the lipid compositions used in this protocol, the average diameters of t- and v-liposomes are ~70 nm (Fig. 2) and the dispersity of the liposomes is monodispersed (PDI < 0.3).

**Figure 3:**
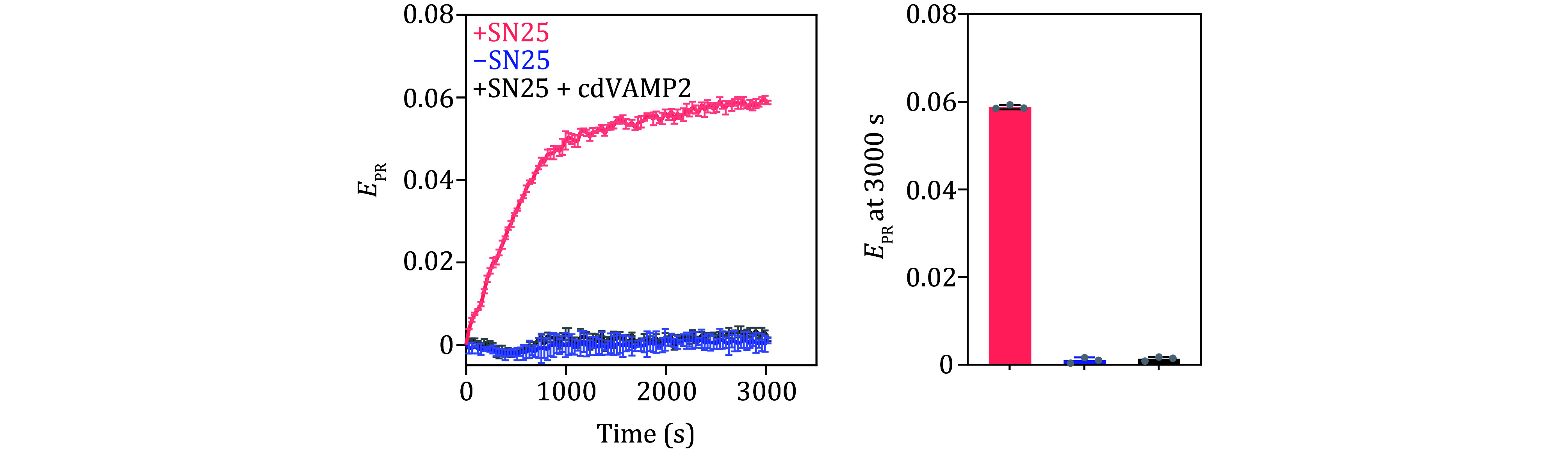
Typical results of SNARE-mediated liposome fusion assay obtained in our reconstitution experiments. Liposome fusion between t-liposomes harboring Syx1 and v-liposomes harboring VAMP2 with the addition of SN25. Data are presented as mean ± SD

Robust and efficient liposome fusion can be observed in the presence of SN25 (Fig. 3). In contrast, no liposome fusions are observed in the absence of SN25 or with excess cdVAMP2, confirming that the liposome fusion is driven by the formation of cognate SNARE complex (Fig. 3). In addition, liposome fusion between t- and v-liposome in the presence of SN25 could be described by single-exponential function, where the time constant (*τ*) and rate constant (*k*) are 591.3 ± 8.1 s and 0.0017 ± 0.0002 s^−1^, respectively.

The above result obtained by the setup in this protocol is only an example. One can optimize certain conditions to meet the requirement of specific experiments, *e*.*g*., adjust the concentrations of soluble proteins, the concentrations of proteoliposomes, the protein-to-lipid ratios, or involve other proteins/macromolecules/chemicals of interest in the system. More examples of typical results can be found in our previously published papers (Liu *et al.* 2023; Wang and Ma 2022; Zhang *et al.* 2023).

## Conflict of interest

Shen Wang and Cong Ma declare that they have no conflict of interest.
